# Traumatic brain injuries: a neuropsychological review

**DOI:** 10.3389/fnbeh.2024.1326115

**Published:** 2024-10-08

**Authors:** Aldrich Chan, Jason Ouyang, Kristina Nguyen, Aaliyah Jones, Sophia Basso, Ryan Karasik

**Affiliations:** ^1^Graduate School of Education and Psychology, Pepperdine University, Los Angeles, CA, United States; ^2^Center for Neuropsychology and Consciousness, Miami, FL, United States

**Keywords:** traumatic brain injury (TBI), neuropsychology, review, assessment, head injury, trauma, neuroscience, psychology

## Abstract

The best predictor of functional outcome in victims of traumatic brain injury (TBI) is a neuropsychological evaluation. An exponential growth of research into TBI has focused on diagnosis and treatment. Extant literature lacks a comprehensive neuropsychological review that is simultaneously scholarly and practical. In response, our group included, and went beyond a general overview of TBI's, which commonly include definition, types, severity, and pathophysiology. We incorporate reasons behind the use of particular neuroimaging techniques, as well as the most recent findings on common neuropsychological assessments conducted in TBI cases, and their relationship to outcome. In addition, we include tables outlining estimated recovery trajectories of different age groups, their risk factors and we encompass phenomenological studies, further covering the range of existing—promising tools for cognitive rehabilitation/remediation purposes. Finally, we highlight gaps in current research and directions that would be beneficial to pursue.

## Introduction

It is possible to speculate that the non-linear path of evolution and its violent history, may have led to the development of brains that favored protection and recovery from brain injuries. To name a few, human brains are protected by the cranium, underneath which are three core layers of protective membranes, in addition to being immersed in cerebrospinal fluid capable of absorbing impact. Indeed, recovery has been found through neuroplasticity and neurogenesis, which appear to be far more advanced than previously believed. At some point however, there was a trade-off, the advancement of brain structure and function, for a brain more susceptible to damage through impact. This adaptation functioned because it was predicated on the utility of complex cognitive abilities to offset and/or treat the consequences of Traumatic Brain Injuries (TBI). One way this is expressed in modern day is the development of neuropsychological assessments in service of evaluating, predicting, and improving the outcome of a TBI.

While TBIs were likely around since the dawn of humankind, it is also likely that our brains did not evolve to withstand the impact of a car crash. Nonetheless, when viewing incidence rates, it is clear that comparable injuries our ancestors might have sustained (e.g., from falls, sports, or violence) continues to be a major problem. Worldwide, traumatic brain injuries (TBI) constitute one of the leading causes of injury-related deaths and disability (Maas et al., 2022). TBIs are responsible for ~30% of all injury-related deaths in the United States and are a leading cause of mortality and disability (Kaur and Sharma, 2018). Closed-head injuries (CHI) account for about 75% of TBIs, while penetrating head injuries (PHI) account for around 25% (CDC, 2016). There were ~223,125 TBI-related hospitalizations in 2019 and 64,362 TBI-related deaths in 2020 (CDC, 2022). TBI is the leading cause of death and disability in people younger than age 35 in the US (Popescu et al., 2015). Falls lead to nearly half of the TBI-related hospitalizations and are now the leading cause of TBI, overtaking road traffic accidents (Roozenbeek et al., 2013). Firearm-related suicide is the most common cause of TBI related deaths in the US. In the United States, around 1.7 million people suffer TBI, with older adolescents (ages 15–19 years) and older adults (ages 65 years and older) among the most likely to sustain a TBI (Georges and Das, 2023).

Traumatic Brain Injury (TBI) refers to penetrating, blunt, or acceleration/deceleration force-derived craniocerebral injury. TBI often results in cognitive deficits in memory, attention, processing speed, word finding, planning, and problem-solving. From a behavioral perspective, difficulties such as lack of initiative, irritability, and poor temper control may be present. Somatic symptoms may include headaches, dizziness, fatigue, sleep disturbance, poor balance, and coordination. TBI can also result in psychological symptoms (e.g., anxiety and depression). These difficulties usually resolve to some degree, but could persist in many cases, even decades after injury. Thus, ongoing assessment and tailored interventions are crucial for effectively managing TBI. Neuropsychological assessments are essential for identifying deficits and understanding the extent of functional loss. These assessments predict outcomes and guide treatment, aiming to improve patients' functional abilities while mitigating further cognitive decline. The specific characteristics of a TBI, including the nature and extent of the damage, help determine the type of neuropsychological deficits that may arise. Understanding these characteristics will assist in distinguishing between different forms of head injuries and their impacts.

## Types of traumatic brain injuries

Head injuries can be classified into two broad categories: closed head injury (CHI) and penetrating head injury (PHI) (Kaur and Sharma, 2018). CHI is more common and complicated than PHI; over 75% of all brain injuries are CHIs. CHI occurs when an external force impacts the skull, causing damage to the brain without penetrating the skull. Swift forward or backward movement and shaking of the brain inside the cranium are common causes of this type of damage, which results in hemorrhage and the tearing of brain tissue and blood vessels (Vieira et al., 2016). CHIs can affect various areas of the skull, including the frontal bone, temporal bone, parietal bone, and occipital bone, depending on the specific circumstances of the injury (Jeyaraj, 2019). Paradoxically, while the cranium is meant to protect the brain, in certain circumstances, parts of it may cause damage. One such area is the crista galli, a protruding triangular surface rising from the ethmoid bone that plays a role in attaching the dura mater (one of the protective membranes mentioned earlier).

On the other hand, PHI occurs when a foreign object, such as a bullet or a sharp projectile, penetrates the skull and directly damages the brain tissue. It is important to note that PHI can cause significant damage to the brain tissue, as the object can cause both primary and secondary injuries to the brain. Primary and secondary injuries are two distinct phases of TBI that can cause damage to the brain tissue (Ng and Lee, 2019). Primary injury occurs immediately after the traumatic event, caused by the direct physical forces applied to the brain tissue. Secondary injury occurs after the primary injury and can be caused by various processes, including inflammation, oxidative stress, excitotoxicity, and others. These processes may worsen the symptoms of the initial injury and inflict more harm on the brain tissue. Secondary injuries can occur over a period of hours or days after the initial trauma and can contribute significantly to the long-term effects of TBI.

CHI can range in severity from mild concussions to severe TBI. In mild cases, the individual may experience symptoms such as headaches, dizziness, and confusion but may not lose consciousness. Severe CHI can result in long-term neurological deficits and disability. Symptoms of closed head injuries can include loss of consciousness, memory loss, difficulty concentrating, seizures, and changes in personality or behavior.

In contrast, PHI is typically more severe and life-threatening than CHI. The severity of a PHI depends on the location and extent of the damage caused by the foreign object. In general, PHIs result in more localized brain damage, whereas CHIs may result in diffuse damage to the brain. PHIs may thus yield a neurocognitive profile with more targeted deficits. This may however, be complicated by hemorrhaging, infection, and swelling, further damaging the brain tissue. Treatment for penetrating head injuries often involves surgical removal of the foreign object, followed by intensive medical care to manage the resulting brain damage and complications. Recognizing the distinction between closed and penetrating head injuries provides context regarding the diverse nature of TBI and their specific effects on brain regions, with these varying impacts directly influencing cognitive and functional outcomes.

## Multiple levels of analysis

The short and long-term sequelae of PHIs and CHIs depend on severity and may be analyzed on multiple levels. Linear and rotational acceleration of the brain can result in mild TBI (mTBI) if a significant amount of force is applied. Blennow et al. (2016) have shown that in these instances, the lower sulci located in the frontal, parietal, and temporal lobes receive higher levels of stress and strain induced by TBI. Cortices and white matter tracts also receive the brunt of the damage induced by mTBI. White matter tracts, which send neuronal signals to nearby neurons and are located in subcortical regions, corpus callosum, fornices, and cerebellum, are more prone to damage upon the initial impact of mTBI.

One of the most common symptoms caused by TBI is cerebral edema. This symptom occurs after the injury and is triggered by an inflammatory response (Arulsamy et al., 2018). Cortical swelling is typically increased in the prefrontal and temporal cortices (Linden et al., 2019; Dall'Acqua et al., 2017). Prolonged and sufficient damage from TBI has the potential to induce an inflammatory response (Bigler, 2013; Johnson et al., 2013) with acute swelling potentially leading to chronic secondary injuries (Ma et al., 2016). That is why, when swelling is severe enough, a portion of the skull may be removed (i.e., craniectomy) to enable the inflammatory processes to take their course, and naturally subside without risking further damage to the brain. Compared with controls, once swelling has subsided, many individuals diagnosed with TBI exhibit reduced brain volume in the temporal, hippocampal, and frontal regions (Bigler, 2013).

Upon impact, axonal shearing may occur near the primary site of injury (Govindarajan et al., 2016). Occurring in areas of the brain initially injured, axonal shearing has been identified as a precursor to the buildup of beta-amyloid plaques, apoptosis, and oxidative stress (Ma et al., 2016). Damage to these areas plays a large role in the symptoms that are experienced. TBI has been shown to produce emotional deficits, challenges with working memory, and other executive dysfunctions. The hippocampus has been correlated to memory related processes and aids in the regulation of emotions. Damage to this area caused by TBI can result in decreased memory capacity and emotional functioning. However, studies have shown that promoting neurogenesis within this region can reduce negative symptoms (Peng and Bonaguidi, 2018).

Moderate to severe TBI significantly reduces cortical thickness (Vedung et al., 2022). Differences in cortical thickness in acute and chronic stages of TBI demonstrate how an injury in the frontal-temporal region correlates to neurodegeneration across the hemispheres. For example, a study was conducted comparing the cortical thickness of mTBI patients where a baseline of decreased cortical thickness was established compared to healthy controls (Govindarajan et al., 2016). Research conducted by Govindarajan et al. (2016) demonstrated that cortical thinning is associated with mTBI primarily in the frontal, temporal, and parietal regions. Follow up examinations of cortical thickness in these subjects revealed the thinning had spread to include some areas of the insula and cingulate cortex (Govindarajan et al., 2016). Research has shown that cortical thickness decreases in adolescents who tested positive for TBI in the prefrontal cortex (Linden et al., 2019). Vedung et al. (2022) established a correlation between a decrease in cortical thickness and an increase in TBI symptom severity, in particular, individuals with moderate to severe TBI exhibit increases in cortical thinning compared with control groups. Within their study, Govindarajan et al. (2016) provided treatment for an mTBI subgroup, participants did not show a significant reduction in cortical thickness after obtaining treatment.

Mild TBI also commonly decreases. However, in some instances, sporadic increases in cortical thickness have been shown to occur post-mTBI, but the increase is not significant compared to cortical thinning (Govindarajan et al., 2016). Individuals who have worse outcomes long term, over 3 months, after the initial injury have lower volumes of gray and white matter and increased cortical thickness compared with healthy controls (Dall'Acqua et al., 2017). The subsequent increase of cortical thickness in mTBI patients has not been well-established, but it could indicate an increase in swelling and trauma.

White and gray matter are largely affected by TBI (Vedung et al., 2022; Dall'Acqua et al., 2017). A decrease in white and gray matter volume inhibits homeostasis as these regions are responsible for neuronal communication and processing. The continuous degeneration of white and gray matter is also found in the neuropathology of neurodegenerative diseases suggesting that secondary injuries of TBI correlate to those diseases (Jang et al., 2017). Jang et al. (2017) found a relationship between the degeneration of white and gray matter tracts and Alzheimer's, Subcortical Vascular Dementia, and mixed dementia, with the highest level of white matter degeneration occurring in Subcortical Vascular Dementia and mixed dementia. The varied impact of TBI on specific brain areas results in diverse experiences and outcomes, with differences observed in cortical thickness, white and gray volume, and the manifestation of secondary injuries.

Findings on how TBIs may impact cortical thickness, white and gray matter, major lobes of the brain, the hemispheres and particular regions may orient the clinician toward specific functions that may be anticipated to be impacted. Yet, real-time dysfunction may be more accurately depicted by a network approach, analyzing neurodynamic imbalances between networks within the brain. Recognizing how TBI disrupts these interconnected networks informs the injury's impact on both localized brain functions and broader cognitive processes.

## Neural networks and TBIs

While there are many neural networks, there are three of particular interest, the Default Mode Network (DMN), the Salience Network (SN) and the Central Executive Network (CEN). The DMN is a network related to mind wandering, autobiographical recall, prospection, self referential processing, and social cognition. The SN determines the significance or salience of external or internal stimuli in any particular moment. It also acts as a toggle between the DMN and CEN. The CEN is a network dedicated to goal-directed tasks and executive functioning. Mutual inhibition typifies the relationship between the DMN and CEN (Chan, 2021). These three networks are typically working together in neurodynamic balance, as the individual shifts their focus toward the external world to focus on a task (CEN) or pauses to reflect on themselves and an interaction that occurred (DMN). In TBIs, intra-network and inter-network disruptions result in broad imbalances and cognitive dysfunction.

From the intra-network perspective, Zhou et al. (2012) have shown that mTBI leads to altered connectivity within the Default Mode Network (DMN), marked by reduced connectivity in posterior regions like the posterior cingulate cortex (PCC) and increased connectivity in the medial prefrontal cortex (mPFC). This imbalance between anterior and posterior regions of the DMN was closely linked to deficits in executive function and mental flexibility, suggesting that such network disruptions may be at the root of some cognitive difficulties commonly observed in mTBI patients. The hyperconnectivity within the mPFC demonstrated an inverse relationship with mood related symptoms such as depression and anxiety. It was further interpreted that the mPFC may initially compensate to sustain cognitive abilities; however, over time, this could contribute to the emergence of psychological symptoms like anxiety and depression.

From an inter-network perspective, Liu et al. (2024) note that it is typical for there to be increased dysconnectivity between the DMN and CEN in mTBI, which has been correlated to reduced working memory performance; abnormal coupling between the CEN and SN which has related to increased emotional dysregulation and internetwork irregularities between the SN and DMN, which has led to disinhibition. Their novel insights come from analyzing the neurodynamic imbalances from a temporal perspective. In comparison to healthy controls, the mTBI group spent the most time in a state characterized by reduced connectivity between the DMN and SN (state 1), whose length of time correlated to reduced scores on the Montreal Cognitive Assessment. Significantly less time was found in a state characterized by higher DMN connectivity, and negative correlation between the DMN and SN (state 3) whom the authors hypothesized may relate to reduced social cognitive abilities. Finally, mTBI participants also demonstrated overall reductions in the amount of transitions between networks.

Disruptions in other networks such as the SN and CEN have also been observed in mTBI. Liu et al. (2020) documented that mTBI patients frequently exhibit hyper-connectivity between the DMN and SN, potentially acting as a compensatory strategy to preserve cognitive performance in the wake of injury. However, this hyper-connectivity may eventually become maladaptive, contributing to further network imbalances and cognitive decline over time. The impact of these network disruptions is not confined to isolated cognitive functions. Research by Li et al. (2020), Li F. et al. (2023), Li X. et al. (2023), and Li C. et al. (2023) demonstrates that mTBI can disrupt connectivity across multiple networks, including the DMN, SN, CEN, and SMN. These disruptions are strongly associated with impairments in attention, executive function, and memory, highlighting the role of network integrity in the cognitive recovery process following injury. Specifically, decreased connectivity between the SN and executive control regions, such as the superior frontal gyrus, relate to the challenges mTBI patients face in maintaining cognitive performance.

In parallel, Rigon et al. (2016) found that mTBI leads to significant reductions in inter-hemispheric functional connectivity (FC) within externally oriented networks (EONs) such as the fronto-parietal network (FPN) and executive control network (ECN), rather than within the DMN or sensorimotor network (SMN). These specific disruptions in inter hemispheric connectivity were associated with impairments in visuospatial and organizational skills, as evidenced by poorer performance on the Rey-Osterrieth Complex Figure Test (ROCFT), implicating inter-hemispheric FC within executive control and flexibility (Rigon et al., 2016).

Structural damage, such as diffuse axonal injury (DAI), significantly impairs key neural networks like the DMN, SN, and CEN. DAI, which occurs in about half of all severe head trauma cases, involves extensive white matter damage that is correlated with cognitive deficits (Aquino et al., 2014). The severity of DAI correlates with its location: mild cases typically involve the frontal and temporal lobar white matter, moderate cases affect the corpus callosum, and severe cases extend to the dorsolateral midbrain. This damage to white matter tracts, particularly in regions like the corpus callosum and midbrain, is a primary contributor to the network disruptions observed in mTBI, leading to cognitive impairments (Aquino et al., 2014).

While the previous studies focused on spatial dynamics between and within networks of the brain, Alhourani et al. (2016) explored temporal dynamics, focusing on frequency-specific changes in connectivity following mTBI. Their study found that mTBI reduces alpha band connectivity and generates slow delta waves, both associated with white matter deafferentation and subsequent cognitive impairments. These frequency-specific disruptions, particularly within the DMN, are linked to deficits in higher cognitive functions such as memory and attention, which are commonly reported post-concussion (Alhourani et al., 2016). Additionally, the observed network topology changes, including the loss of inter-hemispheric connections, may be related to DAI's impact on white matter tracts like the corpus callosum, further exacerbating cognitive deficits in mTBI patients (Alhourani et al., 2016).

## Neuropsychological functions and TBIs

In the context of such foundations as what TBIs are, how they are classified, and how the brain is typically impacted from multiple perspectives, specific neuropsychological functions may now be reviewed in depth. Damage to specific areas (i.e., frontal-temporal cortices and hippocampus) produce the deficits associated with sustaining TBI. As the previously mentioned areas are most vulnerable, the following impaired functions discussed are hallmarks of TBI.

Wang et al. (2021) found support through their research that information processing, memory, and attention are impaired. Individuals with TBI show hyperactivation in the prefrontal cortex, which can lead to cognitive fatigue compared with healthy controls (Gillis and Hampstead, 2015). A review by Blennow et al. (2016) found that impairment of the prefrontal cortex presents difficulty concentrating and poor memory. Additional symptoms related to all severity levels of TBI include nausea, dizziness, vomiting, sensitivity to light, and headaches. Mood changes can also be seen in TBI patients such as an increase in irritability. The treatment of cortical and structural areas resulting in improved functioning that had been damaged by TBI reinforces the role the brain regions play in a healthy brain and what processes are disrupted upon injury.

The prefrontal cortex connects to the limbic system and facilitates top-down processing; damage of these connections correlates to deficits in emotional processing (van der Horn et al., 2015). Emotional impairments are common symptoms of TBI and deficits in processing positively correlate with impairments in accurately identifying negative emotions in individuals with TBI (Rosenberg et al., 2015). Individuals may experience decreased emotional responses or inability to control stronger emotions such as anger (Rassovsky et al., 2015). Axonal shearing of white matter tracts is related to a decrease in general processing speed (Boccia et al., 2022; Ferraracci et al., 2021). This disruption may correlate to damage to the prefrontal cortex. Individuals with TBI compared with healthy controls have demonstrated slower processing speeds (Dymowski et al., 2015). In addition, slowed processing speed, induced by TBI, can affect working memory (Gorman et al., 2016).

TBI may also lead to affective disorders, with anxiety, depression, and PTSD being the most common. Anxiety symptoms, irritability, fatigue, and cognitive deficits persist well-beyond 3 months after the initial impact of TBI (Lamontagne et al., 2022; McMahon et al., 2014). Deficits in language and verbal memory occur consistently with TBI (Wang et al., 2021; Ryan et al., 2015b). The duration of these deficits may depend on the age of the individual when they sustained a TBI and the severity classification. Adults aged 18–24 with TBI had language processing return to normal functioning by 6 months post-injury (Coffey et al., 2021). However, other studies suggest that patients/individuals aged 5–15 years may suffer from language deficits for up to 2 years after the initial impact of TBI (Ryan et al., 2015b). The severity of TBI correlates to the severity of symptoms experienced. Age and severity are variables affecting the degree of potential recovery from damage caused to the brain. Continued research in this area could be useful in determining rehabilitation techniques to reduce the duration of deficits after TBI.

## Classifying the severity of TBIs

The severity of a TBI is a significant factor that affects both the outcomes and therapies for patients (Rauchman et al., 2022). The degree of severity may increase the risk for cognitive deficits, motor impairment, and emotional difficulties (depression, anxiety, aggression, impulse control, etc.), both temporarily and permanently (Mckee and Daneshvar, 2015). Understanding the similarities and differences between these severity levels is crucial for accurate diagnosis, prognosis, and potential treatment methods. Table 1 is a table which his typically used to help classify the severity of a brain injury. Neuropsychologists are responsible for determining the length of Post-Traumatic Amnesia (PTA), a state of discontinuous cognitive functioning, classically characterized by anterograde and retrograde amnesia. One common task administered is the Galveston Orientation and Amnesia Test (GOAT), whereby the TBI patient must receive a score of 75 or greater on three consecutive trials on independent days to be determined out of PTA.

**Table 1:** TBI severity, classifications, and characteristics.

	**Mild TBI**	**Moderate TBI**	**Severe TBI**	
Loss of consciousness (LOC)	<30 min (if present)	30 min−24 h	>24 h	Rauchman et al., 2022
Glasgow Coma Scale (GCS)	13–15	9–12	3–8	Williams et al., 2022
Post-traumatic amnesia (PTA)	<24 h	24 h– <7 days	>7 days	French et al., 2015
Prevalence of cases	75–80%	10–15%	<10%	Laccarino et al., 2018; Mckee and Daneshvar, 2015

Imaging tools play a crucial role in determining the severity of TBI and guiding appropriate treatment strategies. These tools provide valuable insights into the structural and functional changes that occur in the brain following an injury (Mckee and Daneshvar, 2015). By visualizing the affected areas and their extent, imaging techniques enable healthcare professionals to accurately assess the severity of TBI, identify potential complications, and monitor the progression of the condition over time. The following table (Table 2) provides a summary of commonly used techniques and their advantages.

**Table 2:** TBI imaging tools.

	**Description**	**When to use**	**Interpretation**	**Strengths**	
Computed tomography (CT)	Utilizes X-rays to produce cross sectional images of the brain.	Acute phase of TBI to identify bleeding or swelling.	Identifies bleeding, swelling, fractures, or other acute injuries in the brain.	Quick, readily available, effective at detecting fractures and acute bleeding.	Lolli et al., 2016; Power et al., 2016; Schweitzer et al., 2019
Magnetic resonance imaging (MRI)	Uses powerful magnetic fields and radio waves to create detailed images of the brain.	Assessing brain structure, detecting subtle changes.	Detects structural abnormalities, contusions, hemorrhages, or diffuse axonal injury in the brain.	Provides excellent anatomical detail, can reveal contusions, hemorrhages, and diffuse axonal injury.	Lee et al., 2021
Diffusion tensor imaging (DTI)	Variant of MRI that focuses on mapping white matter tracts and assessing their integrity.	Assessing white matter connectivity and abnormalities.	Indicates disruptions in white matter tracts and connectivity, providing insights into the extent of brain damage.	Visualizes neural connections, detects white matter damage, helpful in understanding the impact on brain pathways.	Douglas et al., 2015; Mckee and Daneshvar, 2015
Functional MRI (fMRI)	Measures blood flow and oxygenation changes to assess brain activity and connectivity.	Evaluating functional consequences of TBI.	Shows alterations in brain activity, connectivity, and functional consequences of TBI.	Provides insights into brain function, detects activity changes, reveals connectivity disruptions.	Mckee and Daneshvar, 2015; Scheibel, 2017
Positron emission tomography (PET)	Involves injecting a radioactive tracer to measure brain metabolism and blood flow.	Evaluating brain metabolism and activity.	Highlights areas of altered metabolism, decreased activity, or abnormal glucose utilization in the brain.	Reveals metabolic changes, identifies areas of reduced activity or abnormal glucose utilization.	Huang et al., 2022
Single-Photon Emission Computed Tomography (SPECT)	Involves injecting a radioactive tracer to measure brain blood flow and activity.	Assessing cerebral blood flow and brain activity.	Indicates regions with reduced blood flow, abnormal activity, or functional impairments in the brain.	Helps identify regions of decreased perfusion, abnormalities in brain activity, and functional impairments.	Gosset et al., 2022

## Severity of TBIs and neuropsychological profile

The complexity of TBI-related impairments encompasses cognitive deficits, functional limitations, and behavioral changes. Cognitive deficits, including attention, memory, and executive function impairments, pose challenges in cognitive processes. Functional limitations affect individuals' ability to perform daily activities independently, while behavioral changes can have implications for emotional wellbeing and social interactions (Devi et al., 2020). Behavioral problems following TBI present a significant challenge, yet interventions targeting these problems have received limited attention compared with cognitive and functional deficits (Yeates et al., 2017). Treatment approaches primarily focus on addressing the cognitive and functional aspects of TBI, which can have a profound impact on an individual's daily functioning, work performance, and overall quality of life. Various injury-related factors, such as TBI severity, complications, pre-existing injuries to other body regions, and the duration of the injury, influence the manifestation of TBI symptoms (Rabinowitz and Levin, 2014).

Traumatic brain injury ranges from mild, moderate to severe. Mild TBI (mTBI) is more commonly referred to as concussions. Symptoms experienced in mTBI typically do not exceed 3 months, but they tend to subside within 7–10 days. In cases of mild TBI, individuals may experience temporary cognitive impairments, including difficulties with attention, memory, and information processing speed. Additionally, they may encounter mild functional limitations, such as changes in coordination, balance, and fine motor skills. Fortunately, these deficits are usually transient and tend to resolve relatively quickly. Understanding the neurobiological underpinnings of cognitive impairments and emotional changes following TBI provides valuable insights into the mechanisms involved. Research suggests that mTBI can lead to alterations in synaptic function and plasticity. Disruptions in synaptic strength, which refers to the ability of neurons to communicate effectively, can impair neural communication and impact cognitive processes and overall brain function (Witowski et al., 2019). Rapid changes in synaptic strength may be a contributing factor to attention deficits, memory problems, and learning difficulties commonly observed following mTBI.

In moderate TBI cases, cognitive impairments tend to be more pronounced and long lasting, involving attention and memory difficulties, executive function deficits, and reduced information processing speed. Functional impairments in moderate cases may include persistent motor coordination difficulties, challenges in performing activities of daily living, and emotional and behavioral changes. Severe TBI often leads to severe and persistent cognitive impairments affecting multiple domains, such as attention, memory, language, problem-solving, and executive functions. Furthermore, functional impairments in severe cases can manifest as severe physical disabilities, significant limitations in self-care tasks, difficulties with speech and swallowing, and cognitive and behavioral impairments (Mckee and Daneshvar, 2015). TBI also disrupts the balance of neurotransmitters in the brain, leading to alterations in mood regulation and cognitive functioning (Ahmed et al., 2017). Severe TBI can disrupt emotional contagion, making it difficult for individuals to empathize with others' feelings and maintain social relationships (Rushby et al., 2013). This means they may struggle to pick up on non-verbal cues, misunderstand emotional expressions, and react inappropriately in social situations. This impairment is linked to damage in key brain regions responsible for emotional contagion, like the amygdala and prefrontal cortex (Rushby et al., 2013).

When assessing TBI, different approaches can be taken. As previously discussed, imaging tools are a proficient manner in which to examine this condition and see physical damage to the brain. Another approach to gauging the severity of TBI is to specifically look for difficulties with attention, memory, processing speed, working memory, coordination, and executive functioning. Pen and paper, and computer tests that are administered by a trained professional can acquire such information. These tests inform the clinician to what degree the patient may be experiencing cognitive impairments. Deficits in cognitive functioning are seen in acute and chronic phases of TBI (Tsai et al., 2021).

As research accumulates, so does the opportunity exist for the synthesis of literature that may enable a convenient path for more precise neuropsychological testing. As an example, previous meta-analytic work demonstrates that particular neuropsychological functions were strongly correlated with functional outcome. Allanson et al. (2017) found that delayed verbal memory, visuo-spatial construction, set shifting, and generativity particularly stood out as significant predictors of functional outcome. A more recent meta-analysis supports and builds upon these findings (Krynicki et al., 2023), some of which is presented in Table 3.

**Table 3:** TBI cognitive function diagnostic tools.

**Test**	**Research findings**	**Demographic**	**References**
Test of premorbid functioning (TOPF)	The TOPF has a moderate correlation with Full Scale Intelligence Quotient (FSIQ) (*r* = 0.77). TOPF/demographic is a significant predictor of post-injury FSIQ and displays moderate correlation in predicting FSIQ (*R*^2^ = 0.617, *p* < 0.001). TOPF predicted 35% of variance in FSIQ. Premorbid intelligence of note, TOPF underestimated 31% of premorbid functioning and only accurately identified 38%. VCI in the WAIS-IV predicted greater variance (55%), and may be better alternative individuals with TBI who previously had a high average to superior intelligence.	Participants were accepted into the study if they suffered TBI including mild, moderate, and severe (*n* = 155).	Joseph et al., 2019
Test of memory and malingering (TOMM)	TOMM was best in detecting feigning in TBI (sensitivity = 98.4%) when compared to Groningen Effort Test, the b test and DCT.	Systemic Review and meta-analysis. 82/664 identified studies used.	Azeredo et al., 2024
Wechsler Adult Intelligence Scale Fourth Edition (WAIS-IV) or (WISC—for children)	Controlling for effort and malingering, the WAIS IV detects different levels in performance comparing mild/moderate TBI to severe TBI and healthy controls. The severe TBI group performed significantly poorer than the mild/moderate group in processing speed (PSI) (*p* = 0.001) and FSIQ (*p* = 0.026). Severe TBI had significant reductions on all indices (excluding Matrix Reasoning). Mild/moderate TBI compared to controls had significant reductions in Working Memory Index (AR, LN), Processing Speed Index (SS, CD), and perceptual reasoning subtest (BD). Looking specifically at PSI, adequate sensitivity was shown at 89% and poor specificity at 40%.	Clinical populations diagnosed with either mTBI, moderate TBI or severe TBI (*n* = 100).	Carlozzi et al., 2015
Brief Visual Memory Test- Revised (BVMT-R)	Overall classification accuracy: 59%. Perceptual reasoning significantly influenced BVMT-R performance. It is thus important to use the BVMT-R in tandem with other instruments. The test appeared more sensitive to moderate-to-severe TBI than complicated mild TBI.	Clinical participants: 18–75 years, *n* =100, diagnosed with TBI, no significant premorbid neurological illness, developmental disorders, or psychiatric conditions requiring hospitalization	Donders et al., 2022
Rey-Osterrieth Complex Figure (Rey-O)	Performance validity tests (PVTs) embedded in the Rey-O provide adequate levels of sensitivity and specificity and excellent classification for area under the curve (AUC). Results show that there is excellent classification accuracy using the Sugarman logistic regression formula model 1 and 2 (AUC = 0.80, AUC = 0.83) and Lu and colleagues' equation (AUC = 0.84). A cutoff score of ≥0.45 for Sugarman Model 2 provides 54.1% sensitivity and 97.9% specificity. A cutoff score of ≥0.35 for Sugarman Model 1 provides 43.2% sensitivity and 97.9% specificity. A cutoff score of ≤ 50 for Lu equation provides 48.6% sensitivity and 95.7% specificity. The Rey-O has sufficient embedded performance validity tests for TBI populations.	Clinical veteran population that meets criteria for mTBI (*n* = 100).	Ashendorf, 2019
California Verbal Learning Test, Second Edition (CVLT-II)	The moderate-severe TBI group performed significantly worse on recall and recognition (*p* < 0.0006 and *p* < 0.0008) compared to controls. The mTBI group performed significantly worse than the control group (*p* < 0.02). Sensitivity for recall discriminability is 74.42 and 60.47% for recognition discriminability.	Clinical population (*n* = 43) moderate-severe TBI (*n* = 57 mTBI), and non-clinical population (*n* = 100).	Jacobs and Donders, 2007
Wechsler's Memory Scale	Significant differences were found between groups on the five index scores (*p* < 0.0001) and 10 subtests (*p* < 0.0001). Regarding TBI severity level and the 10 subtests, a moderate effect size was found for mild-moderate TBI and large for severe TBI compared to the normative sample.	Clinical population (*n* = 100) diagnosed with mild/moderate TBI (35%) and severe TBI (65%). Included a health control group for further comparison.	Carlozzi et al., 2013
Color-Word Interference Test (CWIT-4), Delis Kaplans Executive Function System (DKEFS) Color Word Stroop Test	The color inhibition/switching tasks detect differences between moderate/severe TBI and mTBI. Moderate/severe TBI group performed significantly worse than mTBI (*p* < 0.03). The results show the switching and inhibition/switching subtests can distinguish between moderate/severe TBI groups and mild uncomplicated TBI/control groups (sensitivity = 0.64, specificity = 0.67). Moderate-severe TBI group show what is considered a significant decrease in selective attention (*p* = 0.008) compared to controls, also known as an increase in the magnitude of selective interference. The moderate-severe TBI also show a significant increase in dimensional imbalance (*p* = 0.028) compared to controls. Results indicate TBI affects speed of processing. Sensory processing is also affected with TBI participants having an increased latency naming color on color neutral words compared to reading color neutral words (*p* = 0.96, *p* < 0.001).	Clinical TBI sample (*n* = 128) and health controls (*n* = 56). Moderate-severe TBI group (*n* = 324) and healthy control group (*n* = 501).	Anderson et al., 2017; Ben-David et al., 2011
Connors' Continuous Performance Test, Third Edition (CPT-III)	Controlling for effort and malingering, TBI severity significantly correlates to four CPT-II domains. They were greatest for commissions (*r* = 0.463, *p* < 0.001) and detectability (*r* = 0.414, *p* < 0.001); with the others being omissions, and variability.	Clinical population diagnosed with mTBI (*n* = 30), moderate TBI (*n* = 12), and severe TBI (*n* = 18) compared to healthy controls (*n* = 30).	Zane et al., 2016
Trail Making Test, A and B (TMT)	The TMT-B (*r* = 0.29; 95% CI 0.17–0.41) had a significant moderate relationship to functional outcomes. The TMT-B was also associated with a person's ability to return to driving (*r* = 0.3890; 95% CI 0.2678–0.5103). Participants were administered the WAIS-IV and other neuropsychological tests. Regression analysis show that motor, processing speed, backwards span, and sustain components are significant predictors of Trails A (*R*^2^ = 0.45, *p* < 0.02). Trails B is significantly predicted by forward span, processing speed, and backward span (*R*^2^ = 0.33, *p* < 0.02). TMT-C reflects deficits commonly found in TBI. The TMT-C is adjusted from the TMT with 15 circles per page that contain letters and numbers, not 25 circles. This is for both parts A and B.	Meta-analysis of 24/720 studies Children and adolescents previously diagnosed with TBI (*n* = 61). Sample includes mild, moderate, and severe cases of TBI.	Krynicki et al., 2023; Thaler et al., 2013
Verbal fluency (FAS/animals)	Effect size increased with severity. Verbal fluency task are strong indicators of executive dysfunction and language abilities. Effect sizes were moderate to large, phonemic fluency (rs = 0.48) and semantic fluency (rs = 0.45)	Systematic review and meta-analysis of TBI in children: 123/1,516 studies. Meta-analytic review in adults: 1,269 participants, 30 studies	Cermak et al., 2021; Henry and Crawford, 2004
Wisconsin Card Sorting Test– 128 Computer Version (WCST-128)	Compared to healthy controls, TBI patients> perseverative errors, and severe TBI > moderate or mild TBI. Participants with TBI were administered the WCST to examine the relationship between test performance and functional outcomes. Results show that the failure to maintain set errors significant predicts occupational outcomes in TBI patients ≥ 1 year post injury (β = 0.40, *p* < 0.05). The WCST (*r* = 0.20; 95% CI 0.02–0.37) was significantly associated with functional outcomes.	Review of 47/312 studies Clinical population diagnosed with severe TBI (*n* = 143) Meta-analysis of 24/720 studies	Gómez-de-Regil, 2020; Beng et al., 2007; Krynicki et al., 2023
Clock Drawing	The Extended Glasgow Outcome Scale (GOS-E) used to assess outcomes in TBI patients, shows. significant negative correlations per each domain of the Clock Drawing Test. The clock face (*r* = −0.552, *p* < 0.001), numbers (*r* = −0.426, *p* < 0.001), and hands (*r* = −0.511, *p* < 0.001). Better performance on the Clock Drawing Tests is correlated to decreased scores on GOS-E with better functional outcomes.	Participants who sustained mTBI (*n* = 102), moderate TBI (*n* = 30), and severe TBI (*n* = 30).	de Guise et al., 2011
Grooved Pegboard	Neuropsychological tests were used to determine their level of predictability of real-world driving behavior. The Grooved Pegboard, with an established mean cut-off of 97.5 s, can significantly predict performance of on the road driving in TBI subjects. Sensitivity is 0.82 and specificity is 0.29.	Subjects were diagnosed with TBI or stroke (*n* = 78).	Aslaksen et al., 2013
Balance Error Scoring System	High content validity for concussed or fatigued. Large effect sizes after concussions (1.00–1.32) and fatigue (0.54–1.86). average errors for concussion: 17, and fatigue, 15.8. It may not be valid when differences are more subtle. Overall, the BESS: moderate- good reliability to assess static balance.	Systematic review, 29 articles.	Bell et al., 2011
Halstead-Reitan Battery	The Halstead-Reitan Battery (HRB) assessed TBI severity non-impact, impact and no mTBI groups. Results show the tactile test on the HRB producing significant results between groups (*p* = 0.02). Within the tactile tests, the Tactile Form Recognition Test produced the only significant results (*p* = 0.02) (Sweeney and Johnson, 2017). An additional research conducted by Loring and Larrabee (2006) revealed seven subtests to have large effect sizes between TBI and non-TBI groups. The largest effect size was found in the Impairment Index (*d* = 1.77) (Loring and Larrabee, 2006).	Clinical population of non-impact TBI (*n* = 60), impact TBI (*n* = 60), and healthy controls non-TBI (*n* = 29) (Sweeney and Johnson, 2017). Brain damaged clinical group (*n* = 35) including a closed head injury subgroup (*n* = 6) and penetrating head subgroup (*n* = 6) (Loring and Larrabee, 2006).	Sweeney and Johnson, 2017; Loring and Larrabee, 2006

TBI impairs various cognitive functions including memory and executive functions. Alongside these cognitive deficits, TBI has been associated with altered affect and subjective emotional states. Forceful trauma may disrupt neural substrates and subsequently neurotransmitters altering emotions (Ahmed et al., 2017). Often, emotional disruptions experienced following TBI are displayed as behaviors and emotional reactions that cannot be accurately classified by the Diagnostic and Statistical Manual for Mental Disorders (DSM-V) (Shields et al., 2015). Decreased executive functioning coupled with the disruption of neurotransmitters as a result of TBI can have profound effects on emotional regulation. Large correlations are shown between emotional regulation and executive functioning self and informant report forms in an acquired brain injury sample of 64% were a result of TBI (Stubberud et al., 2020). Screening for emotional deficits after TBI improves the care clinicians provide. Emotional measures adequate for assessing mood disruptions in TBI populations is provided in Table 4.

**Table 4:** TBI emotional function diagnostic tools.

**Test**	**Research findings**	**Demographic**	**References**
Beck Depression Inventory-II	BDI-II was broken into four subcategories (somatic symptoms, loss of self-worth, affective symptoms and apathy symptoms) with the intent to distinguish apathy from depression. The apathy subscale and a daily activity log filled out by the participants had a significant negative correlation (*p* = 0.009 and *r* = −0.29). As apathy scores from the BDI-II increased, reported frequency of daily activity decreased. Self-worth and apathy also correlated to a separate measurement of apathy (*p* = 0.002 and *r* = 0.32; *p* < 0.001 and *r* = 0.52).	Participants had a TBI or brain lesion and were recruited from an outpatient clinic in Kyoto, Japan.	Ubukata et al., 2021
Beck Anxiety Inventory (BAI)	The BAI was given to an mTBI veteran population alongside the Neurobehavioral Symptom Inventory (NSI). Results show that a score of 11 on the BAI is associated with the upper range of mild anxiety on the NSI with excellent sensitivity (0.9394) and good specificity (0.6333).	Clinical veteran population (*n* = 308) and 95.8% met criteria for concussion after TBI testing (*n* = 364).	Palmer and Palmer, 2021
Millon Clinical Multiaxial Inventory (MCMI)	Sensitivity and specificity of detecting malingering using the MCMI improve when appropriate cutoffs are applied in TBI populations. A cutoff for disclosure ≥ 67, desirability ≤ 54, and debasement ≥ 71, yield 4% false positive error rate and sensitivity of 47% for disclosure, 55% for debasement and 51% for desirability. This increases accuracy of classing half of malingers and only misclassifying 4% of non-malingerers.	Participants diagnosed with TBI (*n* = 108).	Aguerrevere et al., 2011
Minnesota Multiphasic Personality Inventory, Second Edition (MMPI-2)	Determining appropriate cutoffs for the MMPI-2 to optimize the accurate detection of malingering in TBI and other clinical populations. The results show that a Tscore cutoff of >89 in the FBS-r validity scale provides 48% sensitivity for malingers and 96% specificity TBI participants that passed validity measures.	Clinical population (*n* = 147). Participants diagnosed with TBI (*n* = 59).	Schroeder et al., 2012

## Recovery in children, adolescents, adults, and elderly

The trajectory of healing after traumatic brain injury (TBI) is significantly influenced by an individual's developmental stage. These stages—childhood (ages < 10), adolescence (ages 10–17), adulthood (ages 18–64), and late adulthood (ages 65+)—mark distinct phases characterized by differing resilience levels and recovery challenges post-TBI. Tailoring interventions to suit these stages optimizes rehabilitation by addressing specific developmental needs and challenges, ensuring more effective functional outcomes and enhanced quality of life.

Many studies suggest that TBI sustained in early childhood tends to have more profound and persistent effects on neuropsychological, psychosocial, and educational outcomes compared with TBI experienced in later childhood (Treble-Barna et al., 2017; Sariaslan et al., 2016; Wade et al., 2016). Younger age and greater TBI severity are linked to poorer functional outcomes (Treble-Barna et al., 2017; Sariaslan et al., 2016; Wade et al., 2016). Age of injury is a critical factor as young children have an increased vulnerability to diffuse brain injury and the harmful effects that such injury may have on their growth and development (Treble-Barna et al., 2017). It is suggested that skills that are rapidly developing during the phase of injury are more susceptible to compromise. Consequently, childhood TBI may pose an increased risk of enduring impairments. TBI severity is another critical factor in functional outcomes, as greater severity is linked to lifelong impairments (Treble-Barna et al., 2017; Sariaslan et al., 2016; Wade et al., 2016). Children with moderate to severe TBI tend to display poorer functioning in different domains, including academic performance, community engagement, interpersonal behavior, emotional state, and cognitive processing (Wade et al., 2016). Sariaslan et al. (2016) found that the risks of disability pension and psychiatric inpatient hospitalization were increased by 106 and 92%, respectively, for individuals who had experienced moderate to severe TBI during their childhood, compared with their unaffected siblings. Another study indicated that children who sustained a severe TBI during childhood displayed impairments in adaptive functioning, based on neuropsychological tests and interviews, 6.9 years post-injury (Tomar et al., 2018). These children displayed deficits in fluid reasoning and processing speed, which predicted risk of poorer outcomes in adulthood. Deficiencies in fluid reasoning may hinder an individual's ability to generate solutions and adapt their thinking to navigate daily problems. At the same time, a decrease in processing speed may interfere with daily tasks like interpreting verbal orders or dealing with the demands of a rapid work environment (Treble-Barna et al., 2017). Jones et al. (2018) found that predictors of poor cognitive function in children 12 months post-injury include low socioeconomic status, male gender, living in rural areas, and having experienced a non-accidental injury.

Within 1 month post-injury, children with mild TBI experienced minimal changes in cognitive recovery and quality-of-life (QoL) (Jones et al., 2018). However, by the 6-month mark, there was observable progress in behavioral adjustment, reflecting improvements from baseline. Similarly, 12 months post-injury, children displayed significant cognitive, behavioral, and QoL improvements.

Studies on moderate-to-severe TBI tend to be grouped together. Kennedy et al. (2022) found that older age at the time of trauma correlated with increased mortality and unfavorable overall recovery in children with moderate-to-severe TBI at 6 months post-injury. However, other studies have distinguished the differences between moderate and severe TBI. Those with severe TBI at the 6-month mark show a deterioration in the ability to copy or memorize complex visual materials, suggesting an increased susceptibility to challenging perceptual tasks (Recla et al., 2013). At 24 months post-injury, children with moderate TBI often face difficulties with short and long-term memory in both verbal and visual domains (Catroppa and Anderson, 2007). Whereas, those with severe TBI functioning worsened during the first year, as shown by their accelerated growth curves, indicating an increased presence of executive function dysfunction, specifically related to emotional control, inhibition, and working memory (Keenan et al., 2021). Severe TBI symptoms also displayed a secondary worsening at 24 months.

TBI presents with considerable heterogeneity across different individuals, as factors such as the severity of the injury, location of brain damage, age, sex, and pre-existing health conditions can all influence the manifestation and outcome of the injury, leading to highly variable outcomes (Walker et al., 2018).

Studies have found that TBIs during adolescence may be associated with more difficulties in recovery than in young children and adults because adolescence is known as a critical window for plasticity, substantial maturation, and growth (Mulligan et al., 2021). During adolescence, cortical structures undergo extensive remodeling through the process of synaptic pruning, corresponding to a decrease in cortical gray matter, acceleration of myelination of axons, increased axon density, and increased white matter volume. These changes allow the acquisition of higher cognitive functions, such as improved cognitive control, enhanced behavioral regulation, and better social cognition (Mulligan et al., 2021). The development of social skills during adolescence is critical for group membership and connection to others, which can significantly impact psychological wellbeing (Di Battista et al., 2014; Mulligan et al., 2021).

Because adolescence represents a crucial stage in brain development and maturation, any disruption to this process may have far-reaching consequences beyond the initial injury phase, leading to lasting impairment. The long-term effects of adolescent mTBI can involve attention and executive function deficits (Gutiérrez-Ruiz et al., 2022). Gutiérrez-Ruiz et al. (2022) found that adolescents with mTBI showed lower cognitive processing speeds and a decreased capacity for selective and sustained attentional tasks. These adolescents also showed an increase in symptoms related to anxiety, depression, withdrawal, and social issues (Gutiérrez-Ruiz et al., 2022). Adolescents with moderate or severe TBI experience continuous and significant declines in overall quality of life due to the impact of TBI on important domains of life, such as school, career opportunities, work functioning, and social interactions (Mulligan et al., 2021). Moreover, alterations in self-perception were observed, encompassing feelings of inferior intelligence and self-consciousness resulting in diminished social identity while increasing their reliance on others. These findings are similar to research on adult survivors of adolescent TBI, who reported “poorer school performance, greater employment difficulties, poor health-related quality of life (HRQoL), and increased risk of mental health problems” (Di Battista et al., 2014, p. 1).

With TBI recovery in adolescents, Rivara et al. (2011) found that those with mild TBI, 3 months post-injury, showed a minor decline in quality-of-life (QoL). During follow up periods, at 12 and 24 months, these patients exhibited lower QoL scores, reaching statistical significance without reaching clinically significant levels. In another study, Ryan et al. (2015a) found that adolescents with mild TBI at 12 months post-injury exhibited minimal issues with social functioning, and their social abilities remained relatively stable over time. These studies suggest that sustaining mild TBI during adolescence results in more favorable recovery outcomes.

Adolescents with moderate-to-severe TBI, 3 months post-injury, showed a significant reduction in the range of activities, social and community-based, that they could engage in. Additionally, they experienced a reduction in their communication and self-care abilities compared to their initial baseline measurements (Rivara et al., 2011). At 12 months post-injury, adolescents with moderate-to-severe TBI showed improvement in the range of activities they could participate in, although significant impairments still persisted. Additionally, at the 24- month mark, there were some minor improvements; however, their Pediatric Quality of Life Inventory scores remained significantly lower when compared to baseline. Upon further observation, these patients' communication and self-care abilities showed no significant improvements (Rivara et al., 2011). Between the 12- and 24-month post-injury period, adolescents with severe TBI experienced a significant increase in social problems (Ryan et al., 2015a).

The factors associated with the healing trajectory for TBIs in adulthood include patient characteristics, such as a history of recurrent mTBIs, younger age, and greater educational attainment (List et al., 2015; Rabinowitz et al., 2018). List et al. (2015) found that adult patients with recurrent TBIs had a higher prevalence of cognitive deficits for those who had sustained three or more mTBIs compared with those who had experienced 1 to 2 mTBIs.

Recurrent mTBIs are seen as a risk factor that contributes to the development of dementia later in life. In some individuals, mTBI may even lead to an earlier onset of Alzheimer's disease (AD). The increased risk of AD may be due to accelerated neurodegeneration caused by TBI-induced neurotoxic processes, inflammatory processes, and the accumulation of hyperphosphorylated tau. However, younger adult age has been found to be correlated with more rapid improvement than older age (Rabinowitz et al., 2018). Education has also been indicated as a moderating factor in post-injury function. Rabinowitz et al. (2018) found that participants' education level had a significant effect on their processing speed (PS), executive function (EF), and verbal learning (VL) but did not have an impact on their recovery trajectory. In comparison, younger age was correlated with better recovery of both simple and complex PS and EF. This may indicate that age is a protective factor in the recovery of TBI. However, recurrent injury leads to dose-dependent cortical thinning, resulting in detrimental effects on cognitive function that can contribute to the development of AD (List et al., 2015).

With TBI recovery in adults, Othman et al. (2022) found that 19.2% of adults with mild TBI, 3 months post-injury, showed cognitive impairment, while 19.2% exhibited neuropsychiatric manifestations. At 6 months post-injury, patients continued to experience persistent cognitive impairment, while the remaining majority showed signs of recovery. In another study, Scholten et al. (2015) found that at 6 and 7 months post-injury, adults with mild TBI exhibited significantly more favorable outcomes in various domains, such as Physical Component Summary (PCS) scores, physical functioning, role physical, social functioning, and role emotional. At 12 months post-injury, patients demonstrated outcomes that were similar to population norms, indicating a substantial level of recovery.

In adults with moderate TBI, 3 months post-injury, 39.3% of patients presented cognitive impairment, while 25% exhibited neuropsychiatric manifestations (Othman et al., 2022). At 6 months post-injury, no patients exhibited persistent cognitive impairment or neuropsychiatric manifestations. However, the study by Scholten et al. (2015) found that functional outcome and Health-Related Quality of Life (HRQL) were notably lower when compared to the outcomes observed after mild TBI. Notably, when examined 12 months post-injury, patients with moderate TBI scored higher on physical functioning, general health, and vitality on the Short-Form Health Survey (SF-36; reflecting physical, mental, and social functioning) than those with mild TBI. However, Glasgow Outcome Scale Extended (GOSE) scores were significantly lower than for those with mild TBI (Scholten et al., 2015).

Wilkins et al. (2019) found that in adults with severe TBI, 3–6 months post injury, 43% of survivors demonstrate improvement in their outcome, transitioning from unfavorable to favorable. Interestingly, 6 months post-injury, severe TBI patients scored higher in nearly all SF-36 domains and reported higher PQoL scores than moderate TBI (Scholten et al., 2015). This could be attributed to survivors of severe injuries perceiving some of their challenges as less difficult or their gratitude for being alive potentially outweighing concerns about their functional abilities. However, their functional outcome and HRQL were notably lower compared to the outcomes observed after mild TBI (Scholten et al., 2015). Twelve months post-injury, patients with severe TBI had significantly lower outcomes than mild TBI in the SF-36 domains of PCS, physical functioning, role physical, social functioning, and role emotional. Additionally, GOSE was significantly lower for severe TBI than mild TBI (Scholten et al., 2015). This observation aligns with the study by Wilkins et al. (2019), which reported that 38% of patients with severe TBI improved from unfavorable to favorable outcomes from 12 to 24 months.

When examining traumatic brain injuries (TBIs) in late adulthood, it becomes apparent that the healing trajectory is worse. The majority of the literature indicates an increase in mortality rates and poor functional outcomes. Elderly adults experience worse outcomes after TBIs due to their age, which is found to be an independent risk factor (Prasad et al., 2018; Toth et al., 2021). As individuals age, the brain undergoes atrophy, which leads to an increase in the distance between the brain and skull, making the dural vessels more susceptible to shearing damage. Many elderly patients may live with medical conditions that are masked by TBIs, and their decreased cerebral reserve makes them more vulnerable to minor injuries (Prasad et al., 2018). For instance, if an individual with advanced dementia suffers a head injury, it can lead to cognitive impairments that may prevent independent living. There is a lack of clear guidelines for the treatment of elderly TBI, and the existing guidelines are primarily based on studies of younger adults. This contributes to higher mortality rates and poor functional outcomes in elderly patients. Prasad et al. (2018) further found that, compared with younger patients, elderly patients typically have more extended rehabilitation stays, higher total rehabilitation costs, and a lower rate of improvement in functional measures. Aging and TBI also increase the risk of developing cerebral microbleeds (CMBs). Toth et al. (2021) found that both aging and TBIs can cause CMBs through mechanisms such as cerebrovascular oxidative stress, matrix metalloproteinase activation, and changes in the cerebrovascular wall. CMBs impact the healing trajectory of the elderly because they may lead to “cognitive impairments, psychiatric disorders, and gait dysfunction” (p. 1).

The prognosis for recovery following traumatic brain injury in older adults is generally poor, with substantial evidence indicating high mortality rates and limited functional independence. TBI recovery in older adults shows that those with mild TBI, 3 months post injury, exhibited lower cognitive functioning and performed worse on neuropsychological tests compared to non-injured peers (Hume et al., 2023). At 6 months post-injury, 14% of older adult patients with mild TBI died (Utomo et al., 2009). Thompson et al. (2020) compared older and younger adults with mild TBI, finding that from 1 week to 1 year post-injury, older adults consistently reported poorer overall physical Health-Related Quality of Life (HRQOL) than their younger counterparts.

Older adults with moderate TBI, 3 months post-injury, experience significantly poorer functional health status, with an average Glasgow Outcome Scale Extended (GOSE) score of 5.1, indicating a greater degree of disability following a moderate TBI compared to younger adults (Thompson et al., 2020). At 6 months post-injury, 47.8% of patients with moderate TBI died (Utomo et al., 2009). One-year post-injury, older adults with moderate TBI continued to report an ongoing average of 3.9 symptoms out of 17 symptoms assessed, with a higher likelihood of experiencing balance and coordination issues (Thompson et al., 2020).

**Figure 1:**
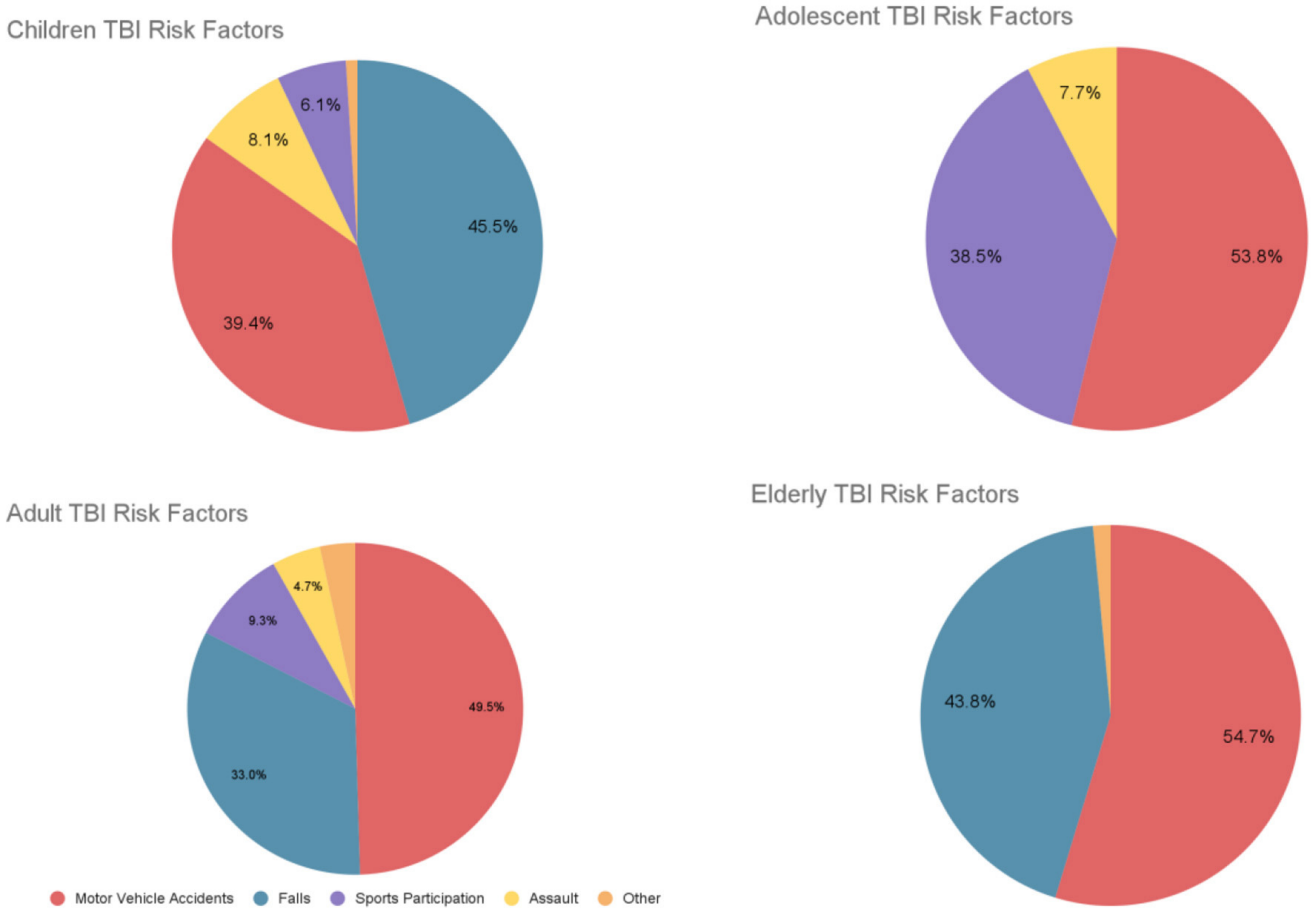
Children, adolescents, adults, and elderly TBI risk factors. Based on data from Keenan et al. (2021), Mulligan et al. (2021), Scholten et al. (2015), and Prasad et al. (2018).

Older adults with severe TBI, 6 months post-injury, with a Glasgow score of <9 exhibited unfavorable outcomes, with a significant mortality rate of 83.3% (Utomo et al., 2009). Similarly, Maiden et al. (2020) found that 6 months post-injury, 85% had died, 47% were living dependently, and 6% had recovered to functional independence. By 12 months post injury, 86.3% had died, 6.4% were living dependently, and 7.3% had recovered to functional independence. Overall, the data reveals that older adults with severe TBI face a substantial risk of death, with only a minority regaining functional independence. The following figure (Figure 1) shows children, adolescent, adult, and elderly TBI risk factors.

## Phenomenology of TBIs

Phenomenological studies of TBI healing progression can provide valuable insights into the subjective experiences of individuals with TBI and the factors that shape their recovery journey. By understanding the individual's perspective on their recovery, healthcare professionals can better tailor their treatment approaches and support the individual in their efforts to recover and return to their everyday lives.

Many phenomenological studies of TBI have interviewed patients during their recovery to assess their functional outcomes and wellbeing over time. Wellbeing is typically categorized as psychological, physical, and social, but there is often overlap between these categories in the literature on TBI healing progression.

Visser et al. (2021) conducted a study examining patients' wellbeing after injury, from their experiences in the emergency room to discharge and rehabilitation. Patients who were severely injured reported feeling a fear of dying while being treated in the ambulance and emergency room. For instance, one patient (female, >16 years) feared she was going to die after seeing blood spouting from her leg, thinking it was arterial bleeding (Visser et al., 2021). Patients who were sedated, unconscious, or experiencing post-traumatic amnesia during treatment often reported feelings of confusion and anxiety about the events of their injury. The realization that they had survived the injury often came to patients during hospitalization and recovery, and this realization was frequently accompanied by a fear of permanent physical limitation, replacing the earlier fear of dying. These findings highlight the initial emotional turmoil and evolving fears faced by patients after severe injuries.

Many patients reported experiencing symptoms of acute stress disorder (ASD) during hospitalization and post-traumatic stress disorder (PTSD) during rehabilitation. Stenberg et al. (2022) followed up with patients 1 and 7 years after TBI and found that wellbeing resulted from adaptation to a recovered or changed life situation. Those with moderate to severe disabilities reported poor wellbeing because adaptation was an ongoing process. However, patients reported that adaptation and wellbeing were facilitated by factors such as leading a purposeful daily life, maintaining an optimistic perspective, and employing adaptive strategies, such as applying knowledge gained from previous hardships. For instance, when asked about the ability to adapt, one patient (Female, 36 years) expressed that despite her permanent injuries, she focused on choosing to be positive and appreciating the good things in life rather than being sad about the negative things (Stenberg et al., 2022). Developing resilience and a positive outlook helped mitigate the negative emotional impact of TBIs in patients' lives.

Experiences related to physical function also contributed to patients' wellbeing. Visser et al. (2021) found that patients often reported that the time needed for recovery was much longer than they anticipated. The desire to feel autonomous rather than helpless often motivated severely injured patients to push through physical limitations during rehabilitation. Stenberg et al. (2022) found that patients with severe TBI reported suffering from life long limitations that impact their health and wellbeing. Overcoming obstacles such as the denial of one's disabilities, feelings of guilt, shame, loneliness, and isolation, and avoidant behavior were all described as necessary for adaptation and wellbeing by severely injured patients. For instance, when asked about difficulties in adapting, one patient (Male, 27 years) expressed increasing depression and frustration due to the persistent impact of his injuries on his life (Stenberg et al., 2022). These experiences reveal the persistent challenges and emotional struggles that accompany physical recovery.

Social support is another critical aspect of TBI recovery, as it is known to improve mental health and overall wellbeing. Visser et al. (2021) found that while it was challenging to rely on others, patients were thankful for the assistance they received from loved ones. In addition, patients believed that their friends' and family's support could aid their recovery. Stenberg et al. (2022) found that living with a severe disability impacted patients' social wellbeing. Patients coping with a severe disability described it as frustrating and leading to loneliness in daily life, as it resulted in exclusion from work and social networks, and the inability to participate in leisure activities. For instance, when asked about living with a disability, one patient (Male, 57 years) described feeling extremely lonely and frustrated by his inability to communicate and participate in activities, ultimately accepting his situation with difficulty (Stenberg et al., 2022). Social support is crucial in mitigating the isolation and emotional distress experienced by TBI patients.

## Defense or deficit?

Phenomenological studies help “humanize” TBI accounts, especially in such reviews as this. The difficulties mentioned in previous examples, in combination with cognitive deficits summarized throughout lead to another important consideration for all neuropsychologists, especially when involved in therapeutic intervention (directly or indirectly). This is the development of the knowledge and skills necessary to help determine whether the patient's presentation is a psychological defense or deficit. These defenses can be a false positive, masking a cognitive deficit, or the cognitive deficit may mimic a defense. The following are some examples where defenses and deficits may be confused:

1. Anosagnosia, or a lack of insight into ones own impairments, may be confused with denial.
2. Executive dysfunction may lead to misattribution of blame, disinhibition and lack of self insight which could be confused with projection, acting out or displacement.
3. Rationalization may also stem from impaired judgment due to frontal compromise, as opposed to a personality type, or emotional reaction.
4. Repression or lack of details related to PTSD could be confused with significant reductions in memory processes related to TBI.
5. Reaction formation or when a polarization of belief occurs, specifically one that was opposite to what the individual previously identified with could arise from deficits in memory, and emotion related systems.

In these instances, a neuropsychological evaluation will play an important role for clinical judgment. TBI victims in inpatient units are typically physically and emotionally vulnerable, creating an environment conducive to the expression of defenses. Outpatients will likely be easier to determine, given discharge is normally associated with a level of stability that improves the likelihood of a comprehensive evaluation, consistency and increased number of sessions.

## Treatment and rehabilitation

Once the patient has been assessed, and deficits ascertained, what can be done? Treatment and rehabilitation for TBI is dependent on the recovery stage of the patient. In the acute stage, in the hours immediately following injury, the main focus of treatment is to stabilize the patient and focus on preventing further injury. Typically, treatment is carried out at an intensive care unit, followed by a neurosurgical ward. For chronic stages of recovery, rehabilitation is the main treatment, and a variety of rehabilitation and treatment protocols exist.

The Rancho Los Amigos Scale-Revised (RLAS-R) is an assessment tool used to describe the cognitive and behavioral patterns found in brain injury patients as they recover from injury. The scale consists of ten levels, with the first level representing the lowest level of functioning, and the tenth level representing the highest. As a patient progresses through higher levels, they demonstrate an improved capacity toward achieving greater independence. Overall, the scale helps facilitate communication among treating healthcare professionals and aids in treatment planning; individualized treatment interventions can be administered depending on the patient's level of functioning and impairments. While individuals move through the different levels in a sequential pattern, the amount of time spent in each level and the maximum level achieved varies by individual. Additionally, levels can be skipped during their recovery, and behaviors can overlap between two different levels. The following table (Table 5) shows the RLAS-R.

**Table 5:** Rancho Los Amigos (revised) (Hagen et al., 1972; Lin and Wroten, 2023).

**Level**	**Cognitive response/need of assistance**	**Clinical features**
Level I	No response/total assistance	No response to external stimuli
Level II	Generalized response/total assistance	Respond inconsistently and non-purposefully to external stimuli. Responses are often the same regardless of the stimulus applied.
Level III	Localized response/total assistance	Respond inconsistently and specifically to external stimuli. Responses are directly related to the stimulus. Tend to be more responsive to familiar people (friends and family) than to strangers
Level IV	Confused and agitated/maxassist	In a hyperactive state with bizarre and non-purposeful behavior. Demonstrate agitated behavior that originates more from internal confusion than the external environment
Level V	Confused, inappropriate non agitated/max assist	Show an increase in consistency with following and responding to simple commands, their responses are non-purposeful and random to more complex commands. Behavior and verbalization are often inappropriate, and the patient can appear confused and often confabulates. Can perform an action or task if it is first modeled or demonstrated for them, they do not yet initiate tasks on their own. Memory is severely impaired and learning new information is difficult. Can show agitation to unpleasant external stimuli
Level VI	Confused, appropriate/mod assist	Able to follow simple commands consistently. Able to retain learning for familiar tasks they performed pre-injury (brushing teeth, washing face) but are unable to retain learning for new tasks. Demonstrate an increased awareness of self, situation, and their environment but are unaware of any specific impairments and safety concerns. Responses may be incorrect secondary to memory impairments but appropriate to the situation
Level VII	Automatic, appropriate/min assist for ADLs	Oriented in familiar settings. Able to perform a daily routine automatically with absent to minimal confusion Demonstrate carry over for new tasks and learning in addition to familiar tasks. Can be superficially aware of diagnosis but unaware of specific impairments. Continue to demonstrate a lack of insight, decreased judgment and safety awareness. Beginning to show interest in social and recreational activities in structured settings. Require at least minimal supervision for learning and safety purposes
Level VIII	Purposeful, appropriate/stand by assist	Consistently oriented to person, place, and time. Can independently carry out familiar tasks in a non-distracting environment. Beginning to show awareness of their specific impairments and how they interfere with tasks, but they still require stand by assistance with compensatory skills. Able to use assistive memory devices to recall a daily schedule. Acknowledge other people's emotional states and require only minimal assistance to respond appropriately. Demonstrate improvement of memory and ability to consolidate past and future event. Often depressed, irritable, and demonstrate a low threshold to rustration
Level IX	Purposeful, appropriate/stand by assist on request	Able to shift between different tasks and complete them independently. Aware of and acknowledge their impairments when they interfere with tasks. Able to use compensatory strategies to cope. Able to independently anticipate obstacles that may arise secondary to any lingering impairments. Able to consider the consequences of actions and decisions with assistance. Continue to demonstrate depression and low frustration thresholds
Level X	Purposeful, appropriate/modified independent	Able to multitask in many different environments with extra time for task completion or devices to assist. Able to create their own methods and tools for memory retention. Can independently anticipate obstacles that may occur as a result of their impairments and take corrective actions. Able to independently make decisions and act appropriately but may require more time or compensatory strategies. May still demonstrate intermittent periods of depression and a lowered threshold for frustration when under stress. Able to appropriately interact with others in social situations

The RLAS-R is typically paired with the GCS to assist in determining the TBI patient's responsiveness. However, unlike the GCS, the RLAS-R is usually used throughout the recovery period, and is not limited to the initial assessment. Earlier screening of cognitive function is important for the prediction of recovery outcomes and facilitates rehabilitation planning.

The administration of the RLAS-R can also be used in conjunction with the Montreal Cognitive Assessment (MoCA). The MoCA is a cognitive assessment that is administered in roughly 10 min and scored on a maximum of 30 points. The domains of function measured includes visuospatial/executive functioning, memory, language, attention, concentration, working memory, and orientation. Individuals with mild and moderate TBI typically have better performances on the MoCA than those with severe TBI (de Guise et al., 2013), and the MoCA can reliably detect impairment in mild TBI and differentiate cognitive disabilities between mild to severe TBI (Mishra et al., 2020).

Once a patient is medically stable following the TBI, they may be transferred to a subacute rehabilitation unit of a medical center, or to an independent rehabilitation hospital. Typically, a multidisciplinary approach is utilized to optimize patient outcomes. The target of rehabilitation treatment will depend on the injury sustained and the associated neurological deficits; as such, treatment may include (but not limited to) physiotherapy, speech and language therapy, cognitive rehabilitation therapy, and occupational therapy.

Cognitive rehabilitation typically consists of training various aspects of cognitive functioning, including learning and memory, visual attention and auditory attention, psychomotor function, and executive functioning. Restorative interventions aim to restore impaired functionality, while compensatory interventions (i.e., cognitive remediation) assist in the development of strategies to minimize the functional impact of the impairment. Neuropsychologists can assess for TBI-induced deficits utilizing a wide-variety of assessment tools, and can prescribe various forms of training to restore or remediate cognitive functioning.

Attention-related difficulties are common among individuals with TBI and can manifest in a variety of ways. Computer-mediated tasks designed to retrain attention can lead to gains on the trained tasks with generalization to similar cognitive measures. However, the results do not always generalize to everyday attentional behavior. Engaging in metacognition, which includes self-reflective capacities and an increase in self-awareness and self-monitoring, has been found to compensate for attentional problems in adults (Ponsford et al., 2014). Metacognitive can also help with problem solving in adults (Tate et al., 2014).

Memory problems can be compensated for in various ways; internal memory strategies, such as mnemonics, visual imagery, and self-instructional methods have been shown to enhance performance on neuropsychological tests. Other helpful memory supports include diaries, notebooks, smart phones, and electronic calendars. Additionally, self-instructional methods that aim to enhance the individual's understanding of manifestations of their memory problems have been shown to improve prospective memory (Velikonja et al., 2014).

TBI often results in communication disturbances that can lead to impaired social competence for both children and adults. Social communication training in group formats has been found to be an effective intervention for individuals 6–24 months post injury. Such training can incorporate everyday communication with partners, and individuals are encouraged to practice the skills they learn beyond the period of training. Social communication interventions can be provided in the natural environment of the person's everyday life (Togher et al., 2014).

Several non-invasive brain stimulation technologies have been developed that can lead to reduced symptomology for TBI patients. The most common technologies include transcranial magnetic stimulation (TMS) and transcranial direct current stimulation (tDCS). tDCS has been shown to reduce TBI-associated depression, tinnitus, neglect, memory deficits, and attention disorders (Dhaliwal et al., 2015). Repetitive transcranial magnetic stimulation (rTMS) is another non-invasive, easily operated treatment that has been shown to improve depression and increase cognitive function after TBI (Neville et al., 2015).

Virtual Reality (VR) has been shown to be an effective rehabilitation tool and can function both as an assessment instrument and as a therapeutic intervention. Dahdah et al. (2017) found that VR can enhance executive functions and information processing in the sub-acute phase of TBI. It has also been used for attention training in severe TBI (Dvorkin et al., 2013), and has been effective in addressing and treating balance deficits (Cuthbert et al., 2014).

Digital brain games overall have yielded mixed results. Some however have demonstrated to efficiently improve cognition among individuals with TBI. BrainHQ is a common and frequently used online platform in which users can play games to train their memory, attention, cognitive speed, interpersonal skills, intelligence, and navigation. Research has found that BrainHQ training can result in both objective and subjective improvements on cognitive measures, which correlates with changes in functional connectivity between the DMN and other resting-state networks in adults with chronic TBI (Lindsey et al., 2022).

Functional electrical stimulation (FES) refers to a process whereby a low-frequency pulse current is used to stimulate limb or organ dysfunction, thereby replacing or correcting lost function in limbs and organs. FES has been shown to have a positive effect on a TBI patient suffering from dysphagia (Calabrò et al., 2016). Though this study only included one individual, the results demonstrated that FES led to significantly improved swallowing functionality in this patient, who could eventually eat solid food safely after the treatment. This suggests that FES may constitute a promising effective treatment for improving lost functionality after TBI, and future research should continue to investigate its potential therapeutic effects with larger sample sizes. There is also evidence that FES can lead to cortical reorganization among individuals with TBI. Milosevic et al. (2021) found that FES treatment led to cortical reorganization and motor improvements in a male participant with chronic TBI suffering from mild motor impairment affecting his right upper-limb. Widespread changes were observed in the motor, premotor, sensory, and parietal cortices in both contralateral and ipsilateral hemispheres and his drawing test performance showed improvements after the intervention and during 3-month follow ups. Again, while this study only included one individual, the results suggest that FES may be an effective tool to improve motor functionality and elicit cortical re-organization and highlight the necessity for future research with larger cohorts of patients.

Hyperbaric Oxygen Therapy (HBOT) refers to the inhalation of 100% at higher than atmospheric pressure and has been used as a treatment for a variety of neurological conditions. HBOT can lead to greatly improved symptomology and a reduction in TBI-related cognitive deficits (Harch et al., 2012). In severe TBI, HBOT has been shown to reduce mortality and enhance functional outcomes in both adults (Lv et al., 2011) and children (Prakash et al., 2012). There is also evidence that HBOT may suppress activation of inflammation signals which TBI induces (Meng et al., 2016). However, it must be noted that despite the published literature supporting HBOT's efficacy for TBI, there have been limited double-blind placebo-controlled trials, and thus HBOT is not an FDA-approved therapy for TBI.

Severe TBI can lead to cerebral edema, which can precipitate intracranial hypertension. The treatment of cerebral edema involves cerebrospinal fluid (CSF) drainage, administration of osmolar agents, and/or craniectomy. The prevention of the development of severe oedema can be targeted by emerging treatments such as glibenclamide, which selectively acts on specific ion channel openings in the brain that can prevent the build-up of fluid (Blennow et al., 2016).

## Future considerations

Future research on traumatic brain injury (TBI) should prioritize the development of multidimensional outcome assessments to more accurately capture individual impairments and establish precise prognostic endpoints. Traditional global or unidimensional instruments may not fully capture the nuances of TBI-related disabilities. Cognitive impairments and mental health outcomes distinguish between patients with TBI on different levels of disability severity. Cognitive impairment often distinguishes between those with moderate and severe disabilities, while adverse mental health outcomes differentiate patients with higher levels of functional outcomes. Multidimensional outcome assessment can utilize unidimensional instruments such as the Rivermead Post-Concussion Symptoms Questionnaire by reconfiguring a general factor of severity of symptoms into core dimensions of psychopathology (e.g., internalizing factors, somatic symptoms) (Nelson et al., 2021). The individual impact of TBI is inextricably related to individual factors (e.g., preinjury mental health, coping skills), as well as the larger socioeconomic context of access to healthcare. Future studies warrant sample cohorts that are large and heterogeneous in order to distinguish factors and contribute toward a multifactorial (bio-psycho-socio-ecological) model of TBI. The multifactorial model, encompassing biological, psychological, interpersonal, and contextual dynamics, clarifies the relationship between individual and socio-economic determinants of health. This model views health as a dynamic interaction of various systems evolving over time. Following a TBI, biological responses like immune system activation shift alongside changes in interpersonal and psychological dynamics. For instance, in the acute phase, biological factors such as inflammation may dominate, while psychological factors like coping strategies become central during rehabilitation (Lehman et al., 2017). Interpersonal dynamics, such as family support, also play a pivotal role throughout the recovery process.

Neuropsychological research should continue distinguishing the efficacy of particular assessments in distinct contexts (e.g.. sex, education, culture, age, etc.). This promotes a more effective selection of batteries that can be tailored to patients. It will also help direct novices in the field in knowing what variables to pay attention to. In addition, approaching TBIs from a neurophenomenological approach may be greatly beneficial. For instance, in major traumatic brain injuries, global dysfunction (e.g., PTA) undergoes a particular temporal sequence toward recovery. This sequence will vary depending on individual differences, yet it is also possible for there to be underlying commonalities more deeply embedded in the brains organic recovery process. A potential sequence may be reflected in a temporospatial model that tracks the development and maturation of emotion and thoughts (Chan et al., 2022). A more nuanced understanding of this sequence may yield important results that may enrich treatment.

Research on TBI should also increasingly address the chronic nature of TBI symptoms given that TBI symptoms can persist or worsen over time and that a majority of moderate to severe TBI patients experience a decline in functioning and an estimated 20 percent die within 5 years of injury (Whiteneck et al., 2018). An individual's functional outcomes at 2 and 5 years after an injury are greatly influenced by their functional status at the first year. However, several factors, such as age, race, payor source (referring to the source of funding for healthcare expenses), length of rehabilitation stay, and cognitive and motor scores, also have a significant impact on how their condition progresses over time (Dams-O'Connor et al., 2020). Current TBI care in the USA frequently fails to meet the needs of individuals, families, and communities impacted by TBI (National Academies of Sciences, 2022). Long-term problems can arise beyond the period of post-acute care: fatigue, sleep disturbance, cognitive impairments affecting memory, attention, and executive function, anxiety, and depression, which can affect individuals' ability to work, study, and drive for at least 5–10 years post-injury (Maas et al., 2022). Although international guidelines for treatment of cognitive and communication impairments have been updated in 2022, more large-scale controlled studies are required to validate new interventions (Bayley et al., 2023).

Furthermore, future research should explore the prevalence and long-term effects of post concussion-like symptoms in patients with different types of injuries. Post-concussion-like symptoms are common among all injury patients, and not just those with head injuries. Understanding the risk factors for developing post-concussion symptoms, such as low educational level, road traffic accidents, chronic diseases, and hospitalization, is also important for early detection and prevention (van der Vlegel et al., 2021). Further studies should also investigate the impact and treatment of post-concussion-like symptoms on the quality of life of patients, healthcare utilization, and return to work rates, as these factors can inform the development of supportive care programs.

The use of biomarkers in classifying injury severity of TBI shows a promising path toward identifying specific patient groups related to disease endotypes. Recent research has assessed certain endotypes (e.g., pH, lactate, blood glucose, and platelet count) (Åkerlund et al., 2022), which may correlate with established measures of TBI impact such as the Glasgow Coma Scale score and degree of metabolic derangement. Other circulating biomarkers like amyloid beta (Hossain et al., 2020) and heart fatty acid binding protein (Lagerstedt et al., 2020) are also being studied to be able to categorize subgroups of TBI patients with different inflammatory endotypes or detect the presence of autoantibodies against brain and extracranial antigens (Maas et al., 2022). As these biomarkers may reflect the body's inflammatory response post-injury, clinicians can more appropriately select and implement anti-inflammatory and immunomodulatory therapies with this data. Future identification of new biomarkers will involve advanced analytic techniques, including artificial intelligence and machine learning, and may help refine important medical decisions throughout the treatment and rehabilitation process. The use of fMRI (Madhavan et al., 2019) and MR spectroscopy (Nadel et al., 2021) as research techniques show promise as future clinical tools for assessing TBI patients. Advanced MRI techniques in combination with volumetric analyses (Stein et al., 2021) have demonstrated increased sensitivity to detecting specific injuries, e.g., traumatic axonal injury, which would typically not be detectable by visual inspection. For mTBI patients, such injuries may not present on CT imagery but are relevant for both persistent post-concussion symptoms and long-term disability. There is also an increasing interest in utilizing MRI to assess the chronic phase of TBI because brain injury biomarkers identified by MRI techniques may predict late brain volume loss and reflect accelerated brain aging (Gan et al., 2021; Yin et al., 2019).

While the field of neurorehabilitation is growing, there is still a lack of support concerning what combinations of treatments may be best depending on the type and severity of TBI. Moreover, the use of computerized interventions has also been on the rise, yet research appears to be mixed. Further specificity as to how these differ from each other may lead to the development of more efficacious programs. Beyond programs focused on cognitive restoration, progress on the development of new compensatory strategies have also appeared to stagnate. It will be beneficial for the development of new strategies considering how much science and technology have advanced (e.g., since birth of ideas such as chunking, visualization or method of the loci). In addition, while individualizing such techniques for everyday experiences is commonly done by practitioners involved with neurorehabilitation, it would be of benefit to identify their efficacy under different contexts. The future of rehabilitation will likely be combined with technological advancements. A deeper examination of roles between clinician and technology will give way to the optimal use of new technologies. An example of this might be the importance of the clinician assisting patients in the translation of skills from virtual brain games to everyday life.

Another route of investigation, more biologically oriented relates to mechanisms underlying the long-term effects of TBI, including the role of neurotoxic processes, inflammatory processes, and the accumulation of hyperphosphorylated tau in the development of dementia and AD. Additionally, more research is needed to understand the psychological effects of TBI, including the prevalence and long-term effects of affective disorders such as anxiety, depression, and PTSD. TBIs can have significant psychological consequences, as they can disrupt the brain's functioning and alter neural pathways involved in emotional regulation. This research may involve longitudinal studies that track individuals with TBIs over an extended period, assessing their psychological wellbeing and identifying risk factors that contribute to the development of affective disorders. By gaining a better understanding of these psychological effects, healthcare professionals can develop more targeted interventions and support systems to improve the overall outcomes and quality of life for individuals with TBIs.

## Conclusion

The assessment and treatment of TBIs provides a unique window into the brain's capabilities and vulnerabilities. Similar to how brain lesions provide a deeper understanding of their corresponding functions, TBIs may be a lens into the underlying processes of cognition, emotion and personality. TBIs are a multidimensional, complex injuries that can result in a range of physical, cognitive, and emotional deficits. The brain areas most vulnerable to injury are the frontal and temporal cortices leading to deficits in executive functioning, attention, memory, language, and emotional processing. Deficits in processing correlate with impairments in identifying negative emotions, affective disorders such as anxiety, depression, and PTSD. Mild TBI, accounting for the majority of cases, can cause temporary post-concussive symptoms that may resolve within a few months and temporary cognitive symptoms within a few days. However, even a single mTBI or recurrent mTBI can result in the emergence of depressive-like behaviors up to 90 days following the injury. On the other hand, moderate and severe TBIs are more serious and can lead to long-term complications, even death.

The trajectory of healing for TBI varies depending on the developmental stage of the individual. Adolescents who sustain TBI may have more difficulties in recovery than young children and adults due to their critical window for plasticity, substantial maturation, and growth. Several factors influence the healing process of traumatic brain injuries (TBIs) in adults. These factors include patient characteristics like a history of repeated TBIs, younger age, and higher levels of education. Individuals who have experienced multiple TBIs are more likely to have cognitive deficits compared with those who have had fewer incidents. Recurrent TBIs are also associated with an increased risk of developing dementia later in life. In certain cases, mTBI may even accelerate the onset of Alzheimer's disease.

Evolution has left humans with the cognitive ability to generate sophisticated methods for the assessment and treatment of TBIs. Grounded in research, neuropsychological assessments are themselves a product of evolutionary necessity, offsetting our brains vulnerability by providing critical and nuanced information that can be used practically. At its core, neuropsychological results provide (1) viable predictions on functional outcome that can assist with expectation management, (2) strengths that can be used to compensate for challenges, (3) weaknesses that are specifically targeted for restoration/compensation, and (4) an optimal path for recovery and adaptation.
